# On Syphilitic Ulcers of the Larynx

**Published:** 1823-05

**Authors:** Cæsar H. Hawkins

**Affiliations:** Member of the Royal College of Surgeons.


THE LONDON

### Medical and Physical Journal.

5 OF VOL. XLIX.] MAY, 1823. [No. 291.

For many fortunate discoveries in medicine, and for the detection of numerous errors, the world is indebted to the rapid circulation of Monthly Journals; and there never existed any work, to which the Faculty, in Europe and America, were under deeper obligations, than to the Medical and Physical Journal of London, now forming a long, but an invaluable, series.—RUSH.

### ORIGINAL COMMUNICATIONS,

### SELECT OBSERVATIONS, &c.

ART. I.—*On Syphilitic Ulcers of the Larynx.* By CÆSAR H. HAWKINS, Member of the Royal College of Surgeons.

[Concluded from page 281.]

SYMPTOMS of consumption are sometimes produced by ulcers in the larynx and trachea, the lungs being at the same time healthy. To this disease the name of Phthisis Laryngea has been given; a long-continued cough, with purulent expectoration, gradually exhausting the patient, the suppuration having reduced him to the most extreme state of emaciation. The ulceration in such cases sometimes extends down the trachea, and even into the bifurcation of the bronchiæ; the whole of the inner membrane being irregularly studded with ulcers, having a honeycomb appearance; even the rings of the trachea are sometimes eroded and sloughy. The great extent of the disease in such cases may perhaps arise from the chronic nature of the ulceration, which is not severe enough to excite those symptoms of ulceration of the glottis, so often fatal before those of consumption commence.

It is asserted by some persons that it is most difficult, or altogether impossible, to distinguish between phthisis pulmonalis and phthisis laryngea. The cough, the purulent discharge, the emaciation, the hectic, are, it is true, common to both diseases; but still, I think, there are some marks by which they may be known. These are to be found in the peculiar form of the chest of consumptive persons; in the extent to which the patient has the power of expanding his lungs, which is almost always limited by the alteration of their structure, which takes place in phthisis pulmonalis; in the sound emitted from the chest by percussion, the change of tone being commonly perceptible by simple tapping with the fingers, even without the assistance of the stethoscope, to which so much importance is at present attached. Add to which the fact, that tapping the ribs gently will almost always excite coughing in persons whose lungs are unhealthy, while sound lungs will scarcely be affected by it.

I have thus endeavoured to point out some of the most characteristic marks of ulceration of the glottis. I have done this, perhaps, at greater length than some persons may think the occasion deserves, and dwelt too much on points of minor interest; but I believe it is only by attention to these minutiæ that an accurate diagnosis can be established, the correctness of which must be of the last importance; for the treatment, which will be successful in some of the diseases of the larynx, will be useless or prejudicial in others, which are apparently of similar character.

In the first few cases of ulcerated larynx which I saw, although the existence of the ulcer was known or suspected, the symptoms resembled so much those of cynanche laryngea, that the same means were resorted to which are usually employed with success for that disease. I have seen leeches and blisters applied repeatedly in the same persons; I have bled to syncope; I have given calomel to salivation, according to Dr. Cheyne's recommendation; and no progress has been made towards the cure. Recourse was then had to the means presently to be mentioned; and the same patients, who had suffered frequent relapses for the two or three preceding weeks, have become in as many days nearly convalescent.

Possibly, too, on the first inspection of a throat covered with ulcers, perhaps syphilitic in their appearance, or connected with a very suspicious history, an inexperienced practitioner might be tempted to employ mercury; and that, perhaps, introduced with a rapidity proportionate to the urgency of the symptoms.

The *acute sloughing ulcer*, which was first described, is of such a nature that it will often be fatal under the most judicious treatment. The depression of the whole system is so excessive, that the most powerful stimuli may fail to rouse the constitution: and, although they seem to be the only means to which we can have recourse, their exhibition requires perhaps considerable caution and judgment; for the inflammation of the throat itself is very high, while the general system is in the lowest state of debility; and there is so much local irritability, that the passage of medicine and food may of itself be sufficient to increase the rapidity of the sloughing.

The best method, therefore, appears to be, to endeavour to soothe the parts, (for which purpose the steam from hemlock-leaves will often be found effectual,) while we support the system rather by nutritious diet than by strong cordials. Ammonia is of great service in such cases, and it may be given at the same time with sarsaparilla; which latter medicine is, perhaps, one of the most powerful means we possess for the support of the strength, independent of any antisyphilitic virtues which it may boast of. The best form in which we can exhibit it with this intention, is perhaps the syrup, which can be made of greater strength than the usual preparations, and acts more speedily upon the constitution; nor, perhaps, are the highly nutritious qualities of the sugar to be lost sight of. Its chief fault is a liability to offend the stomach; the tendency to which will, however, generally be counteracted by the addition of a little tincture of ginger, or some other aromatic. After the desired effect has been obtained, and the violence of the disease checked, some other form of the medicine, or some tonic, may be substituted for the syrup of sarsaparilla.

The second species of ulcer to be mentioned, is that to which I have given the name of *the chronic sloughing ulcer of the throat.* There are few diseases, so formidable in appearance, which are so completely under the control of medicine, as this ulcer is in most cases. As an illustration of the treatment proper in such cases, and of the powerful agency of our curative means, I will mention the case of a person who had an ulcer of this description, which had destroyed the tonsils and uvula and part of the soft palate, and occupied the whole of the posterior part and sides of the pharynx, as high up in the nares as could be seen, the lower part reaching below where the membrane is visible. He had become much emaciated during the three preceding months, and had an irritable pulse, beating 140 times in a minute, with nocturnal perspirations and very great prostration of strength. He took a pint of the Lisbon diet-drink, and fumigated his throat twice a-day with the red sulphuret of mercury. In ten days, his pulse had sunk to 70, and had become quite soft and regular; his throat was very nearly cicatrized, and he had already gained flesh considerably. Other stimulants, such as the linimentum æruginis, or tinct. benz. c., may be substituted for the fumigations, if they are not thought advisable; but I think no internal medicine can be employed with the same advantage as the sarsaparilla.

I have seen too little of *the painful ulcer of the throat,* to recommend any thing with confidence from my own experience. Two cases, which I have heard described, were cured by mercury, even although the disease had in one of them extended to the glottis. There are, however, so few sloughing sores with which mercury agrees, and the risk, if it does not suit any individual ulcer, is so considerable, that at least the utmost degree of attention should be paid to its effects. I should rather be inclined to trust the cure to very large doses of solid opium, or at least delay the employment of mercury till the excessive pain is relieved, which is alone sufficient to keep up that state of the system on which the sloughing of a sore depends. It has already been mentioned, that the difficulty of swallowing is very great, so that life has in some cases been sustained chiefly by nourishing enemata.

Many persons have doubted the possibility of our effecting a cure, if the ulceration has once extended to the larynx itself. This opinion appears to me, however, to be erroneous, as, in one or two instances which I have examined, I have seen what had all the appearances of cicatrices from former ulcerations. This was particularly observable in the larynx of a woman who had all the usual symptoms of ulcerated larynx in a severe degree, and was discharged cured. She continued well for several months, and was then re-admitted into the Lock Hospital, with a return of the symptoms, accompanied with phthisis pulmonalis; of which she died. An ulcer was seen in one side of the glottis, with a ragged depression in another part of the larynx, which was probably the remains of the previous attack. Judging from their resemblance in feeling to some fatal cases, where dissection had verified the observation previously made with the finger, I believe that I have felt ulceration of the epiglottis and sides of the chordæ vocales, in persons who have been afterwards cured. In one very remarkable case, after the symptoms of ulcerated larynx had subsided, the cicatrix seemed to have so far contracted as nearly to close the glottis, producing very great difficulty of breathing, with a noise evidently showing the small size of the tube through which the air had to pass. The greatest relief was obtained by the occurrence of fresh ulceration in the lower part of the pharynx, which appeared to have in some measure re-opened the cicatrix of the former ulceration.

The most dangerous symptoms attending ulceration of the larynx, have been stated to arise from the difficulty of breathing; and this takes place, not from any actual impediment to the passage of the air through the larynx, but from a spasmodic action of the muscles of the glottis, produced by the ulcers in the vicinity. The consequence of this difficult respiration is the excessive debility of the patient, from the insufficient supply of arterial blood: and here, perhaps, the prostration of strength and death of the patients do not arise so much from want of nutrition, as from the circulation of dark venous blood through the body, by which the brain and nervous system become oppressed. For the emaciation of the patient is not always in proportion to the extreme degree of languor and debility under which he labours; and in one case the patient died in convulsions, as if from an affection of the brain.

This view of the subject will easily account for the inefficacy of common antiphlogistic treatment in this stage of the disease;* for, if the symptoms are for a time relieved by them, the actual cause, namely, the ulcer in the larynx, remains unaffected and incurable by these means. The symptoms will therefore return again and again, till the cause is removed.

There are, therefore, two objects which we ought to aim at in the management of ulcerated glottis: the first is, to relieve the excessive irritation produced by the ulcer, which threatens to destroy our patient, before much progress can be made in the healing of the ulcer itself; and, having thus in some measure ameliorated the most urgent symptoms, the cure of the ulceration itself must next engross our attention.

Several plans may be tried in order to gain the first object; such as inhaling the vapour of a strong infusion of conium or opium, or of nitric acid diluted with warm water. If the ulcer can be touched with the finger, the solution of lunar caustic may be applied, in the manner recommended by Mr. Charles Bell,† which appears to perform the same part here that it does in severe cases of spasmodic stricture of the urethra. It does not act as an escharotic, but simply diminishes the nervous irritability on which the contraction of the muscles depends. On the whole, however, I believe that fumigations of cinnabar are by far the most powerful means we possess of effecting this purpose. Very great relief is often experienced from the first time this plan is resorted to. Half-a-drachm or a drachm of the red sulphuret of mercury may be used once or twice a-day, according to the facility with which it is borne, or the benefit that is derived from it. The mercurial fumigation is not employed with a view to its specific action upon the system, for its effects are equally obvious when the constitution is not at all affected by it, as when the gums are made sore by its employment: salivation, indeed, to any great degree, would be disadvantageous, and perhaps dangerous. The action of the cinnabar rather appears to be local, though it would perhaps be difficult to explain exactly the manner in which it acts.

It must be obvious that the use of the two latter remedies are chiefly applicable to those ulcers which follow the chronic ulcer of the throat; and that the soothing plan is most appropriate in the more acute and inflammatory species, while the violent local action exists.

As a few hours are often of consequence, we may call in the aid of leeches and blisters to the throat, or even of general bleeding, if circumstances require it; for, although these means

---

* *Medico-Chirurgical Transactions*, vol. vi. p. 249.

† *Surgical Observations*, part i. p. 36.

are not to be trusted as our principal remedies, they are sometimes very useful auxiliaries.

Having checked the most urgent symptoms by some of the preceding plans, we gain time to effect such a change upon the system as to ensure the healing of the ulcer. For this purpose sarsaparilla may be given, which requires a few days before its virtues are exerted in any sensible degree, and should therefore be generally employed from the beginning, in the manner before mentioned: or, if we wish for a more immediate change in the constitution, we may employ bark in large quantities, the effects of which may be obtained in a few hours. I prefer, however, the less obvious, but more steady, action of sarsaparilla. The employment of ammonia in pretty large doses is of great service in some cases. At the same time, the diet must not be neglected; but it should, in general, be light and nourishing.

After a few days, if the case goes on favourably, a very great change is perceived in the symptoms. The respiration becomes less laborious, and the patient is no longer disturbed in his sleep by convulsive starts; he can swallow without inconvenience; his pulse changes its character; and the colour of the lips and surface of the body returns to a healthy state.

How far the operation of bronchotomy may be successful in cases of ulcerated larynx, is a question of some importance. I have only seen it performed in one case of this kind, which was a disease of the larynx following the acute sloughing ulcer of the throat. The patient was seized with a sudden fit of difficult respiration, having all the appearance of spasmodic action of the muscles of the glottis. He was evidently so nearly dead, (and in fact he died in less than five minutes after the seizure,) that I considered myself justified in opening the larynx immediately, as the only possible means of saving his life. He scarcely lived long enough, however, to breathe through the artificial opening; nor was his recovery to be expected, from the large quantity of frothy mucus with which the lungs were gorged.

In Mr. Lawrence's paper on the Affections of the Larynx requiring the operation of Bronchotomy,* a number of successful cases are recorded; but almost all of them were performed for the extraction of foreign bodies, or for acute diseases of the larynx; and I do not recollect any successful case, in which ulceration of the larynx was known or suspected to exist, although there are several cases in which the patient's death was evidently retarded.

I cannot but think that the operation is only likely to be attended with success in the more acute diseases of the larynx,

* *Medico-Chirurgical Transactions*, vol. vi. p. 240, 241, *et seq.* and 250.

and is not at all advisable in cases of ulceration. It is allowed on all hands, that the effects of diseases of the larynx "are in themselves fatal after a certain time, even if the original obstruction be obviated:" hence the operation "is ineffectual, unless performed early."* There are very few cases of ulceration in which the operation is likely to be proposed till the disease has existed so long that these fatal effects have probably supervened; and I am inclined to think that all those who would get well after the operation, might be cured without it. Of the two last cases mentioned by Mr. Lawrence, the first appears to me to have been evidently one of inflammation; the last was one of ulcerated larynx, mentioned also by Mr. Bell, who performed the operation: the former succeeded, the latter failed.

Mr. Lawrence remarks, "The different results of Donovan's case, (the ulcerated larynx,) particularly after the favourable appearances exhibited in some parts of its progress, leads us to reflect on the causes of such a difference. Although the operation was longer delayed in the latter instance, and the artificial opening less free, yet the death of the patient must, I think, be ascribed to an original difference in the nature of her affections: a difference which, for the reasons so well pointed out by Dr. Latham, cannot be recognized by the symptoms. We have already seen that different affections are discovered, after death, in patients whose symptoms, derived from the interruption of the respiratory and vocal symptoms, which is common to them all, exhibited no diagnostic differences; that in some there was a mere thickening and change of structure in the membrane, while in others the cartilages were diseased. We may be allowed to conjecture that Jones's disease was of the former kind; while we know, from dissection, that Donovan's was of the latter."† It appears, from these remarks, that Mr. Lawrence would not advise the operation of bronchotomy where the cartilages of the larynx are diseased. It has been my endeavour, in this paper, to point out the diagnostic characters by which this may be known: with what success I have done this, it is not for me to determine.

It would be useless to enlarge upon the more advanced stages of this disease, as, probably, such affections are, in the present state of our knowledge, wholly incurable.

*St. George's Hospital; December* 1822.

---

* *Medico-Chirurgical Transactions*, p. 248.

† *Ib.* p. 262.

